# Feature Engineering for the Prediction of Scoliosis in 5q‐Spinal Muscular Atrophy

**DOI:** 10.1002/jcsm.13599

**Published:** 2024-12-05

**Authors:** Tu‐Lan Vu‐Han, Vikram Sunkara, Rodrigo Bermudez‐Schettino, Jakob Schwechten, Robin Runge, Carsten Perka, Tobias Winkler, Sebastian Pokutta, Claudia Weiß, Matthias Pumberger

**Affiliations:** ^1^ Charité – Universitätsmedizin Berlin, Corporate Member of Freie Universität Berlin and Humboldt‐Universität Zu Berlin, Center for Musculoskeletal Surgery Berlin Germany; ^2^ Explainable AI for Biology Zuse Institute Berlin Berlin Germany; ^3^ Berlin Institute of Health at Charité – Universitätsmedizin Berlin, BIH Biomedical Innovation Academy Berlin Germany; ^4^ Center for Humans and Machines Max‐Planck Institute for Human Development Berlin Germany; ^5^ Institute of Health, Berlin Institute of Health Center for Regenerative Therapies Berlin Germany; ^6^ Interactive Optimization and Learning Zuse Institute Berlin Berlin Germany; ^7^ Department of Pediatric Neurology Charité‐Universitätsmedizin Berlin, Berlin, Germany; Center for Chronically Sick Children, Charité‐Universitätsmedizin Berlin Berlin Germany

**Keywords:** feature engineering, gene therapy, machine learning, predictive power score, rare disease, spinal muscular atrophy

## Abstract

**Background:**

5q‐Spinal muscular atrophy (SMA) is now one of the 5% treatable rare diseases worldwide. As disease‐modifying therapies alter disease progression and patient phenotypes, paediatricians and consulting disciplines face new unknowns in their treatment decisions. Conclusions made from historical patient data sets are now mostly limited, and new approaches are needed to ensure our continued best standard‐of‐care practices for this exceptional patient group. Here, we present a data‐driven machine learning approach to a rare disease data set to predict spinal muscular atrophy (SMA)‐associated scoliosis.

**Methods:**

We collected data from 84 genetically confirmed 5q‐SMA patients who have received novel SMA therapies. We performed expert domain knowledge‐directed feature engineering, correlation and predictive power score (PPS) analyses for feature selection. To test the predictive performance of the selected features, we trained a Random Forest Classifier and evaluated model performance using standard metrics.

**Results:**

The SMA data set consisted of 1304 visits and over 360 variables. We performed feature engineering for variables related to ‘interventions’, ‘devices’, ‘orthosis’, ‘ventilation’, ‘muscle contractures’ and ‘motor milestones’. Through correlation and PPS analysis paired with expert domain knowledge feature selection, we identified relevant features for scoliosis prediction in SMA that included disease progression markers: Hammersmith Functional Motor Scale Expanded ‘HFMSE’ (PPS = 0.27) and 6‐Minute Walk Test ‘6MWT’ scores (PPS = 0.44), ‘age’ (PPS = 0.41) and ‘weight’ (PPS = 0.49), ‘contractures’ (PPS = 0.17), the use of ‘assistive devices’ (PPS = 0.39, ‘ventilation’ (PPS = 0.16) and the presence of ‘gastric tubes’ (PPS = 0.35) in SMA patients. These features were validated using expert domain knowledge and used to train a Random Forest Classifier with an observed accuracy of 0.82 and an average receiver operating characteristic (ROC) area of 0.87.

**Conclusion:**

The introduction of disease‐modifying SMA therapies, followed by the implementation of SMA in newborn screenings, has presented physicians with never‐seen patients. We used feature engineering tools to overcome one of the main challenges when using data‐driven approaches in rare disease data sets. Through predictive modelling of this data, we defined disease progression markers, which are easily assessed during patient visits and can help anticipate scoliosis onset. This highlights the importance of progressive features in the drug‐induced revolution of this rare disease and further supports the ongoing efforts to update the SMA classification. We advocate for the consistent documentation of relevant progression markers, which will serve as a basis for data‐driven models that physicians can use to update their best standard‐of‐care practices.

**List of Abbreviations**
CHOP‐INTEND: children's Hospital of Philadelphia Infant Test of Neuromuscular Disorders
6MWT: 6‐minute walk test
CSV: Comma‐separated value (.csv) file
EHR: Electronic health record
EMA: European Medicines Agency, EU
FDA: Food and Drug Administration, U.S.
HFMSE: Hammersmith Functional Motor Scale Expanded
HINE‐2: Hammersmith Infant Neurological Examination Module 2
ML: Machine learning
NMD: Neuromuscular disease
NMS: Neuromuscular scoliosis
RF: Random Forest Classifier (Scikit‐Learn)
ROM: Range of motion
RULM: Revised upper limb module
RWD: Real‐world data
SMA: Spinal muscular atrophy

## 1 Introduction

### 1.1 Disease‐Modifying Therapies in Spinal Muscular Atrophy (SMA)

Rare diseases are classified as those that affect fewer than 1 in 2000 people in Europe (European Medicines Agency, EU [EMA] definition) and 1 in 200 000 in the United States (Food and Drug Administration [FDA] definition) [1]. Due to the few patients affected, the study of rare diseases poses a challenge to healthcare providers. Only highly specialised centres of excellence, where rare disease cases are often consolidated, have the resources to investigate the field [2]. Concentrating knowledge and expertise is essential to provide these patients with the best up‐to‐date standard of care. However, even specialised physicians face new unknowns when novel disease‐modifying therapies enter the field. With more promising drug targets on the horizon, new approaches to address these unknowns are urgently needed to ensure a continued best standard of care.

5q‐SMA, a rare monogenic neuromuscular disorder with an incidence of approximately 1 in 7000 [3], recently became one of the 5% treatable rare diseases. Based on the natural history of SMA, the current classification distinguishes four SMA subtypes depending on the age of onset, *SMN2*‐gene copy numbers and achieved motor milestones [4]. Type 1 (also known as Werdnig–Hoffmann disease or acute infantile SMA) is the most severe form, affecting infants and leading to early mortality, often within the first 6 months. Types 2 and 3 (also known as Kugelberg–Welander disease or juvenile SMA) manifest in childhood with differing degrees of motor impairment and survival, while Type 4 presents in adulthood with milder symptoms [5]. In 2016, the FDA approved the first SMA therapy, an antisense oligonucleotide called nusinersen [6]. This was followed by FDA approval of the first gene therapy, onasemnogene abeparvovec, in 2019 [7]. The first orally available drug approved by the FDA was risdiplam in 2020 [8]. These therapeutic options have resulted in significantly prolonged survival and improved prognosis in affected SMA patients [7] and led to the implementation of SMA in newborn screenings in nine countries [9], including Germany, to facilitate early diagnosis and treatment [3]. Early treatment administration has demonstrated improved efficacy, likely due to high SMN protein expressions during foetal and postnatal periods, which has led to investigations of potential in utero administration [10]. Despite promising results, SMA therapies are not curative, and the overall developments create a dynamic, heterogeneous SMA patient landscape. These patients now present with scoliosis and other SMA‐associated sequelae for interdisciplinary consultations.

### 1.2 Previous and New Challenges in SMA‐Associated Scoliosis

Scoliosis plays a central role in the disease progression of SMA because it is closely associated with disease severity. Historically, up to 90% of patients develop scoliosis [11]. The disease's underlying progressive degeneration of motoneurons and muscular atrophy often lead to an early‐onset and rapidly progressive spinal deformity, which, if left unaddressed, can lead to thoracic insufficiency syndrome [12]. Previous studies on SMA‐associated scoliosis primarily focused on SMA Types 2 and 3 as the predominant surviving patient groups [12]. Natural history data of the SMA Type 1 patient cohort are especially lacking [13]. In addition, because of the heterogeneity and small number of affected patients, there is a marked lack of expert consensus in managing these early‐onset scoliosis [14]. Drug‐induced new unknowns now aggravate these previously existing challenges.

### 1.3 Data‐Driven Machine Learning (ML) Approaches Could Aid in Treatment Decisions

Previous studies used statistical methods to make inferences based on SMA patient data sets. These methods are easy for physicians to interpret, and the conclusions drawn from a representative sample of patients were used to make treatment decisions. Amid a more dynamic and likely ever‐changing patient landscape, the applicability of these conclusions may now be limited. While statistical methods can observe the relationship between variables, data‐driven ML approaches may be able to find generalizable predictive patterns in the data that can aid in decision‐making on a future unseen data set [15]. Leveraging the power of such an ML‐based approach could help physicians augment their treatment decisions in the era of gene therapies. Here, we explore a data‐driven ML approach to the current challenges in SMA. We used patient variables from multiple specialties and tested whether expert domain knowledge‐driven feature engineering can improve the predictive power for modelling in a rare disease data set. We aimed to lay the groundwork for future rare disease data sets that may undergo a drug‐induced disease evolution.

## 2 Material and Methods

### 2.1 SMAScoliosis Data Collection and Data Set Enrichment

We built a unique SMA patient data set containing clinical parameters of multiple subspecialties routinely involved in treating SMA patients, i.e., neuropediatricians, orthopaedic surgeons, spine surgeons, geneticists and physiotherapists. To ensure the generalizability of our results, the patient parameters used in this study are also routinely collected in the SMArtCare study, the largest European SMA data registry. The physicians and physiotherapists involved in the data collection were trained in the SMArtCare study [16] to ensure uniform and standardised assessment. SMA patient data were collected via electronic health records (EHRs) and Picture Archiving and Communication System (PACS). The database was built using Research Electronic Data Capture (REDCap) tools hosted at Charité Universitätsmedizin Berlin [17, 18]. The data collection instruments were designed to follow the flow of patient examinations during routine visits. Text data were minimised during instrument design to reduce variability and human‐induced error during data collection, and each field was uniquely coded to receive an unambiguous input. To avoid input errors further, we implemented the REDCap input validation tool. To control for potential bias in the data set, we collected paraclinical data, including place of birth, country of origin, patient ethnicity, family history, parental consanguinity and baseline medical history, including the number of co‐existing illnesses and medications. Data collected from PACS included the scoliosis parameters (i.e., radiograph date, Cobb angle, measurement tools) and notes on the quality of radiographs.

### 2.2 Data Export and Preparation

The SMAScoliosis data set was updated entirely and exported in February 2023. The unprocessed data frame collected data from 84 patients with 1304 visits (rows) and over 360 variables (columns). We analysed the data set to test for SMA data set characteristics. Before feature engineering, the data set was cleaned, and the variables were grouped by their associations. We tested the variables for missing data and overall distribution within the data set. Variables with significant amounts of missing data were manually analysed for their type of missingness by persons involved in the data collection. Data missing due to inherent REDCap data collection design (i.e., branching mechanisms) were thereby discerned from missing data due to errors during data collection or data unknown to us. All data processing, feature engineering, analyses and modelling were performed in Python Software Foundation (version 3.11) [19].

#### 2.2.1 Feature Selection Techniques

We used an expert domain knowledge‐driven feature selection and engineering approach, which involved an analysis of the current literature and physicians specialising in treating SMA patients, to select features that correlate with SMA disease progression and SMA‐associated scoliosis development. These features were prioritised over features with unknown or no relevance.

### 2.3 Feature Engineering

**FIGURE 1:**
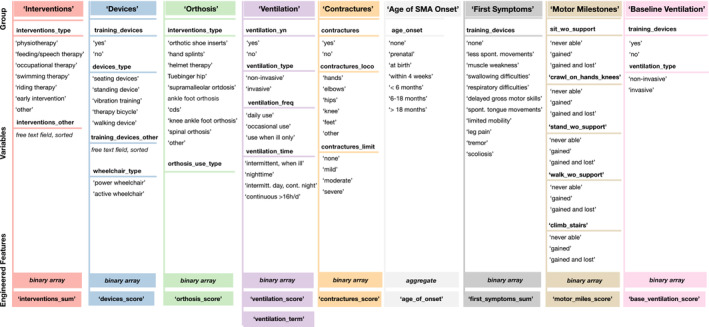
Engineered features from variables of the SMAScoliosis data set. Variables were grouped by association, converted to numeric and then binary arrays or aggregated (grouped). The binary array was used to calculate either a sum or a score. Detailed mathematical equations used for feature engineering are described in the Supporting Information.

Figure 1 shows the groups of variables used to engineer their corresponding features. A detailed description of the feature engineering process for each feature is listed in the Supporting Information. As an example, we will describe the engineering process of the feature ‘contracture score’. The relevance of the localisation of contractures in SMA patients has previously been characterised by Fujak et al. [20, 21], where contractures in the knees and hips occur earlier in SMA disease progression than contractures of the upper limbs. The severity and localisation of contractures have an implicit relevance in disease progression and, thus, scoliosis development. To code this information into a single feature, we performed feature aggregation: We ranked the importance of the contracture localisation in the order of onset and significance as previously described for SMA by Fujak et al. [20, 21]. Next, we converted the ranking into a binary array (loco_i_) and factored in the severity of contracture (contractures_limit_) from mild (1), moderate (2) and severe (3):

$$\begin{array}{c} \begin{array}{c} {\text{contracture}}_{\text{score}}=\sum_{i=1}^{6}{\left(2^{i}\;\times \;{\text{loco}}_{i}\right)}^{\text{contracture}s_{\text{limit}}/3}\;\\ \;{\text{loco}}_{i}\in \left\{0,1\right\},\;\text{contracture}s_{\text{limit}}\in \left\{1,2,3\right\} \end{array} \end{array}$$

We used different feature selection tools, including correlation analysis, predictive power score (PPS) analysis and PPS ranking to analyse the various relationships between the features. All packages are implemented in Python.

#### 2.3.1 Correlation, PPS and PPS Ranking Analysis

To compute the correlation matrix, we used Pandas correlation packages, which use Pearson correlations to calculate correlation coefficients using pairwise complete observations [22]. For data visualisation, we used Seaborn v.0.12 and Matplotlib v.3.6.3 packages [23, 24]. To perform PPS analysis of the SMAScoliosis features, we used Python's ppscore v1.3.0. The PPS is an ‘asymmetric’, data‐type agnostic score that can detect linear or nonlinear relationships between two columns. The score ranges ‘from 0 (no predictive power) to 1 (perfect predictive power)’ [25]. For data visualisation of the PPS matrix, we used Seaborn packages v.0.12 [23]. To rank all of the PPSs for the target label ‘scoliosis’, we used the PPS package (pps.predictors) v1.3.0. First, we looked at the functional motor scores for SMA individually. Next, we calculated each feature's PPS and ranked them by their predictive power from highest to lowest. Results of the PPS analysis and expert domain knowledge guided the final selection of the most relevant features.

### 2.4 Scoliosis Labels

**FIGURE 2:**
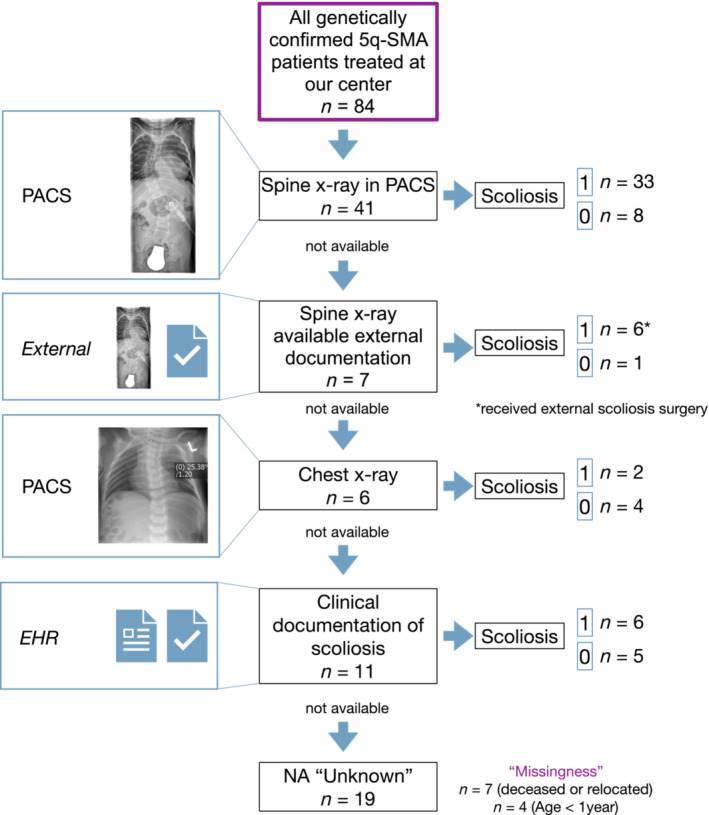
Flow chart data collection of scoliosis labels using the most reliable scoliosis detection method. All patients with genetically confirmed 5q‐SMA were included in the SMAScoliosis data set. Labels were collected from anteroposterior spine radiographs, and the scoliosis label was ‘1’ (positive) if a Cobb angle > 10° was measured and ‘0’ (negative) if a Cobb angle was < 10°. Forty‐one patients had available spinal radiographs in the PACS, and the scoliosis was labelled according to the measured Cobb angle. If no PACS spinal radiograph was available, but external orthopaedic treatment was documented in the patient EHR (i.e., external radiological or orthopaedic report), the scoliosis label was derived from the documentation (7 patients). If no spinal radiograph was available, we used chest radiographs (6 patients) if the observation aligned with the clinical documentation (e.g., ‘clinical exam: orthograde spine’). The scoliosis label was derived only if both corresponded. Next, we derived the scoliosis label from clinical examinations in the patient's EHR. If multiple entries over time documented higher‐grade clinical scoliosis or lack thereof, the label was set accordingly. All other cases were labelled ‘unknown’ (NA).

**FIGURE 3:**
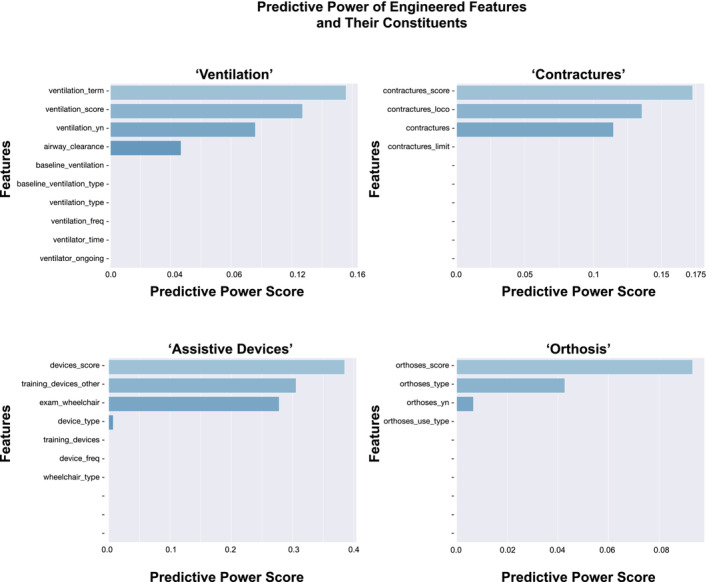
Predictive power score of engineered features versus their constituents. Bar plots grouped by the engineered features ‘Ventilation’, ‘Contractures’, ‘Assistive Devices’ and ‘Orthosis’. The *y*‐axis labels give the constituent features, and the *x*‐axis the predictive power score of the feature to ‘scoliosis_yn’.

The natural history of scoliosis in SMA is progressive upon onset. To label scoliosis at the time of scoliosis detection, we performed a retrospective scoliosis label analysis of all 84 patients (Figure 2). Labels were collected from anteroposterior spine radiographs, and the scoliosis label was ‘1’ (positive) if a Cobb angle > 10° was measured and ‘0’ (negative) if a Cobb angle was < 10°. Forty‐one patients had available spinal radiographs in the PACS, and the scoliosis was labelled according to the measured Cobb angle. If no PACS spinal radiograph was available, but external orthopaedic treatment was documented in the patient EHR (i.e., external radiological or orthopaedic report), the scoliosis label was derived from the documentation. If no spinal radiograph was available, we used chest radiographs if the observation aligned with the clinical documentation (e.g., ‘clinical exam: orthograde spine’) (Figure 3). The scoliosis label was derived only if both corresponded. Next, we derived the scoliosis label from clinical examinations in the patient's EHR. If multiple entries over time documented higher‐grade clinical scoliosis or lack thereof, the label was set accordingly. All other cases were labelled ‘unknown’ (NA) and were not used for training and testing.

### 2.5 Scoliosis Prediction Model

To test the performance of the selected features, we used a Random Forest Classifier (RF) from Scikit‐learn v1.2.1 [26]. The SMAScoliosis data set was cross‐validated and split into train (0.8) and test data (0.2) using a grouped K‐fold cross‐validation strategy, and metrics were averaged over 10 folds. The model performance was evaluated using the standard confusion matrix and average receiver operating characteristics area under the curve (ROC AUC).

## 3 Results

### 3.1 Statistics of the SMAScoliosis Data Set

The SMAScoliosis data set consisted of 84 male patients, 47 (55.95%) and 37 (44.05%) female. Thirty‐two (38.10%) SMA Type I, 31 (36.90%) Type II, 18 (21.43%) Type III and 3 (3.57%) presymptomatic/type unknown patients. At the time of cross‐sectional analysis, 17 (53.13%) of the SMA Type I patients had scoliosis, 21 (67.74%) with SMA Type II and 9 (50.00%) with SMA Type III, respectively. The mean age of onset was 2.02 months (2.41 SD) in the SMA Type I group, 9.95 months (5.79 SD) in the SMA Type II and 42.63 (43.10 SD) in the SMA Type III group. The number of visits per patient ranged from 1 to 19 (mean 5.96, median 5, SD 4.12) (Table 1). Regarding SMA therapies, 23 (27.38%) patients had received nusinersen, 8 (9.52%) risdiplam and 14 (16.67%) onasemnogene abeparvovec monotherapy. Thirteen (15.48%) patients had received a combination of nusinersen and risdiplam and 22 (26.19%) had received a combination of nusinersen and onasemnogene abeparvovec therapy. For four patients (4.76%), the SMA therapy type was unknown (i.e., not documented). Regarding scoliosis labels, of 84 SMA patients, 41 had available spine radiographs in the PACS, of which 33 had a measured Cobb angle > 10° and were labelled with scoliosis = ‘1’. Seven patients received documented external spine radiographs and orthopaedic treatment (six had received scoliosis surgery outside our centre). Chest radiographs were available for six patients, two of whom had visible thoracic scoliosis on the chest radiograph, which aligned with the clinical examination in the patient's EHR. A total of six patients had documented clinical scoliosis, and five had an orthograde spine. The 19 remaining patients received the label ‘NA’ (unknown).

**TABLE 1:** Statistics of the SMAScoliosis data set and corresponding cross‐sectional ‘scoliosis labels’.

*n* = 84	Counts	Gender	SMN2 copies(mean)	SMN2 copies(std)	Mean age‐of‐onset(months)	Age of onset(std)	Min age‐of‐onset(months)	Max age‐of‐onset(months)	Scoliosis label		Scoliosis(%)
Type 1	32	*M* = 19	2.218	0.49	2.02	2.41	Birth	9	Yes	17	53.13%
		*F* = 13							No	8	25.00%
									Unknown	7	21.88%
Type 2	31	*M* = 11	3.107	0.497	9.95	5.79	Birth	24	Yes	21	67.74%
		*F* = 15							No	6	19.35%
									Unknown	4	12.90%
Type 3	18	*M* = 10	3.5	1.095	43.1	43.1	Birth	168	Yes	9	50.00%
		*F* = 8							No	5	27.78%
									Unknown	4	22.22%
Presympt	3	*M* = 2	3	0	None	0	None	n.A.			n.A.
		*F* = 1							Unknown	3	n.A.

*Note:* The data set consists of 84 genetically confirmed 5q‐SMA patients.

### 3.2 Correlation Analysis of SMA Patient Features

**FIGURE 4:**
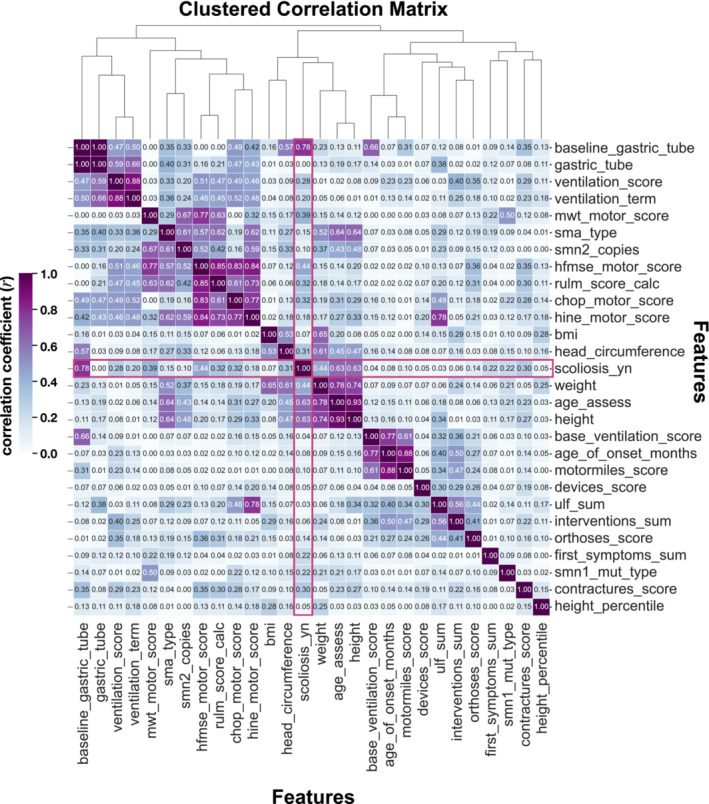
Correlation matrix of features from the SMAScoliosis data set: features are listed on the *x*‐axis and *y*‐axis. The correlation coefficient *r* is annotated accordingly. We observe some positive correlation between correlation matrix, where we observe some positive correlation between ‘baseline gastric tube’ (*r =* 0.78), ‘ventilation score’ (*r =* 0.28), ‘6MWT score’ (*r =* 0.39), ‘HFMSE score’ (*r =* 0.44), ‘RULM score’ (*r =* 0.32), ‘CHOP motor score’ (*r =* 0.32),), ‘head circumference’ (*r =* 0.31), ‘weight’ (*r =* 0.44), ‘age at assessment’ (*r =* 0.63), ‘height’ (*r =* 0.64), ‘first symptoms sum’ (*r =* 0.22) and ‘contractures score’ (*r =* 0.30) with our target label ‘scoliosis’.

We performed a correlation analysis for feature selection and calculated correlation coefficients between features in the SMAScoliosis data set to observe potential positive or negative correlations. The correlation matrix (Figure 4) shows a positive correlation between ‘baseline gastric tube’ (*r* = 0.78), ‘ventilation score’ (*r* = 0.28), ‘6‐minute walk test (6MWT) score’ (*r* = 0.39), Hammersmith Functional Motor Scale Expanded ‘(HFMSE) score’ (*r* = 0.44), revised upper limb module ‘(RULM) score’ (*r* = 0.32), ‘CHOP motor score’ (*r* = 0.32),), ‘head circumference’ (*r* = 0.31), ‘weight’ (*r* = 0.44), ‘age at assessment’ (*r* = 0.63), ‘height’ (r = 0.64), ‘first symptoms sum’ (*r* = 0.22) and ‘contractures score’ (*r* = 0.30) with our target label ‘scoliosis’. Positive correlation coefficients could be observed between known SMA classification markers, ‘SMA type’ and ‘*SMN2* copy numbers’ (*r =* 0.61). The functional motor scales showed an overall positive correlation with one another: ‘CHOP motor score’ and ‘HINE motor score’ (*r =* 0.77), ‘6MWT motor score’ and ‘HFMSE score’ (*r* = 0.77), ‘RULM score’ and ‘HFMSE score’ (*r =* 0.85), respectively. We observed a positive correlation between the ‘baseline ventilation’ and the ‘baseline gastric tube’ (*r =* 0.66). A correlation existed between the patient's number of ‘interventions’ and the ‘orthosis score’ (*r =* 0.41).

### 3.3 PPS Analysis and PPS Ranking of SMA Patient Features

**FIGURE 5:**
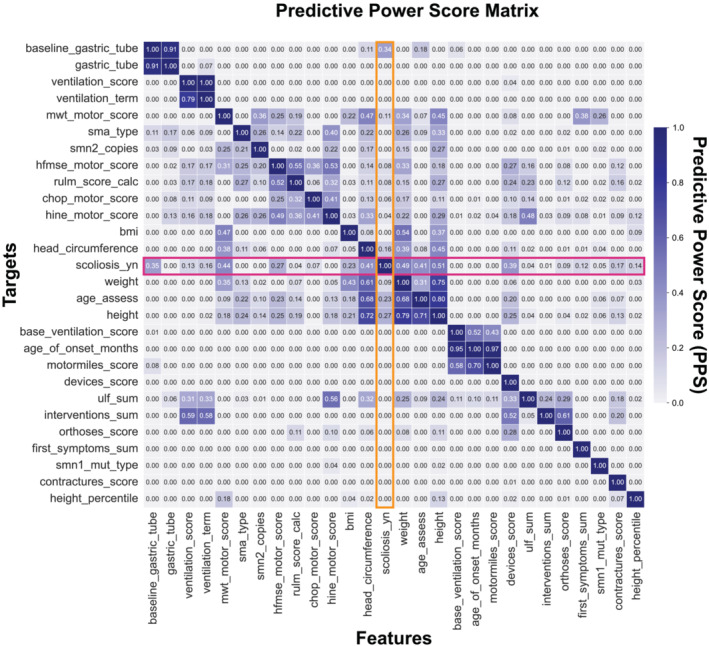
Predictive power score matrix of features from the SMAScoliosis data set: the *x*‐axis lists the features that predict the targets on the *y*‐axis. Predictive power scores are annotated accordingly. Predictors of our target label ‘scoliosis_yn’ (*y*‐axis) include ‘age_assess’ (0.41), ‘baseline_gastric_tube’ (0.35), ‘BMI’ (0.23), ‘contractures_score’ (0.17), ‘devices_score’ (0.39), ‘head_circumference’ (0.41), ‘height’ (0.51), ‘hfmse_motor_score’ (0.27) and ‘6mwt_score’ (0.44 ‘weight’ (0.44).

**FIGURE 6:**
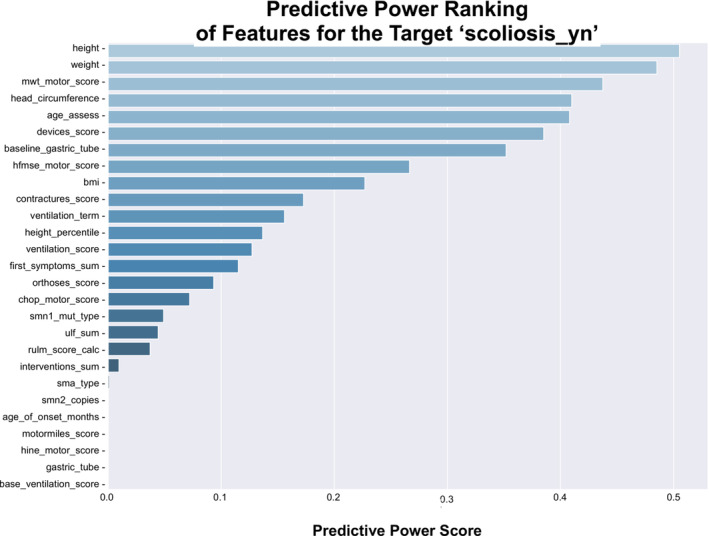
Feature ranking by calculated predictive power score (PPS).

We computed the PPS between features and their respective targets. Figure 5 shows the PPS matrix with the PPS for each feature pair annotated accordingly. Features on the *x*‐axis predict the targets on the *y*‐axis. We observed that predictors of our target label ‘scoliosis’ included ‘baseline gastric tube’ (0.35), ‘ventilation score’ (0.13), ‘ventilation term’ (0.16), ‘6MWT score’ (0.44), ‘HFMSE motor score’ (0.27), ‘BMI’ (0.23), ‘head circumference’ (0.41), ‘weight’ (0.49), ‘age assessment’ (0.41), ‘height’ (0.51), ‘devices score’ (0.39), ‘orthoses score’ (0.09), ‘first symptoms sums’ (0.12), ‘contractures score’ (0.17) and ‘height percentile’ (0.14). In contrast to the correlation matrix (Figure 4), the variables ‘CHOP motor score’ (*r* = 0.32), ‘contractures score’ (*r* = 0.30), ‘first symptoms sum’ (*r* = 0.22), ‘RULM score calc’ (*r* = 0.32), ‘SMA type’ (*r* = 0.20) and ‘*SMN2* copies’ (*r* = 0.3) did not show high predictive power for scoliosis in the PPS matrix. Next, we ranked the features by their PPS for the target label ‘scoliosis’ (Figure 6) to select the most relevant features for scoliosis development. The ranking showed scoliosis predictors including ‘height’, ‘weight’, ‘BMI’, ‘head circumference’ and ‘age at assessment’. Disease progression markers, such as the ‘HFMSE score’ and the ‘6MWT score’, ranked high on PPS. Engineered features ‘devices score’, ‘contractures score’, ‘ventilation score’ and ‘orthoses score’ also had high PPS scores. Notably, disease markers, such as ‘SMA type’, ‘*SMN2* copy numbers’, ‘*SMN1* mutation type’, ‘baseline ventilation’ and ‘age of onset’ did not have high PPS.

### 3.4 Testing Engineered Features Against Their Individual Variables

To compare engineered features with their individual variables, we ranked the PPS (Figure 6). We observed that the scores of our engineered features ‘ventilation term’, ‘ventilation score’, ‘contractures score’, ‘orthosis score’ and ‘devices score’ achieved higher PPS than their individual variables (Figure 3). This emphasises the value of feature engineering during data preparation in small data sets. Other variables that we aggregated, such as ‘age of onset’, ‘first symptoms sum’, ‘motor milestones’ and ‘interventions sum’ did not increase the predictive power. Functional motor scores are related to muscle development and disease progression in SMA and are routinely assessed using validated scores depending on the age and developmental stage of the patient. Because each score consists of multiple single items, we tested whether some individual items performed better than the total score (see Supporting Information). We observed that total scores generally had higher PPS, so we used the total functional motor scores as features.

### 3.5 Testing Selected Features With an RF Prediction Model

**FIGURE 7:**
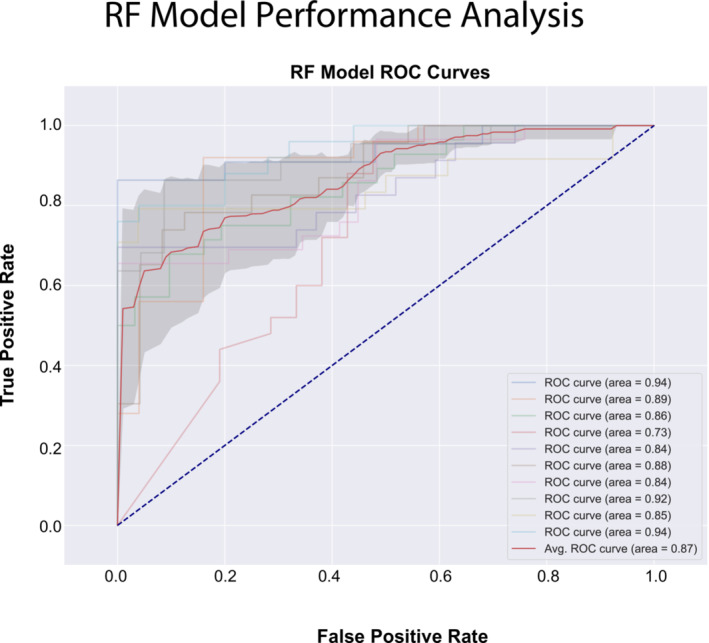
Receiver operating characteristics (ROC)‐curves of a random forest classifier trained with SMAScoliosis features.

To test the general performance of the selected features from the SMAScoliosis data set for the prediction of a binary ‘scoliosis’ classifier, we used a RF (specifically the implementation from Scikit‐Learn [26]). RFs are robust and capable of handling outliers while suitable for small data sets. We trained the model using the features ‘age assessment’, ‘height’, ‘weight’, ‘devices score’, ‘BMI’, ‘head circumference’, ‘baseline gastric tube’, ‘HFMSE motor score’, ‘contractures score’ and ‘ventilation term’; these features seemed the most informative based in the PPS ranking. We performed 10‐fold cross‐validation using a stratified grouped K‐Fold training strategy, and on average, the model achieved an accuracy of 0.82 with a precision of 0.85. Figure 7 (right) shows the ROC AUC. The average ROC AUC was 0.87, with a well‐controlled standard deviation. The performance metrics across all 10 folds are listed in Table S2.

## 4 Discussion

This study applied ML tools to a paediatric rare disease data set to define relevant markers for disease‐associated scoliosis development. We demonstrate the differences in data preparation for a physician's learning versus an ML algorithm and provide critical insights for the field. By applying feature engineering techniques, we overcome one of the main challenges in data‐driven approaches in rare disease data sets: data scarcity. We demonstrate how to develop practical tools to anticipate scoliosis development and improve scoliosis management in these patients. We defined relevant clinical features for scoliosis development using ML predictive power tools. These included paediatric growth parameters, ‘age’, ‘height’, ‘weight’, ‘BMI’, SMA‐specific functional motor scales ‘HFMSE score’, ‘CHOP‐INTEND score’, the presence of ‘contractures’, ‘ventilation’ and the use of ‘devices’ and ‘orthoses’. Using expert domain knowledge, these features have confirmed clinical relevance and are associated with SMA disease progression.

### 4.1 Our Relevant Features Align With a Current Paradigm Shift From Disease Biomarkers to Progression Markers

The current classification of SMA is based on the age of SMA onset, achieved motor milestones, and *SMN2* copy numbers [5]. These markers represent prognostic markers, assessed when the disease is first diagnosed. However, these markers may have low predictive power in a drug‐induced dynamic disease landscape. This can be observed in our ranking of relevant features for scoliosis development (Figure 6), where the age of onset, achieved motor milestones and *SMN2* copy numbers ranked low. Instead, dynamic markers that are reassessed at each visit score high in terms of predictive power. These results align with our previous understanding of SMA‐associated scoliosis development: Previous studies have demonstrated that SMA‐associated scoliosis development is linked to the loss of ambulation [27] and thus to SMA disease progression. In line with this understanding are the results of our study: We show that SMA‐associated scoliosis development is a function of the patient's age and development and known disease progression markers [28, 29, 30], such as ‘HFMSE’, ‘6MWT’ scores and the use of ‘ventilation’, ‘devices’ and ‘orthoses’. These features can train an algorithm to predict scoliosis development. Using these features, we trained a scoliosis classifier, demonstrating how data give rise to meaningful insights that can help physicians identify informative clinical features in a patient to aid diagnostics and improve scoliosis treatment and potentially downstream outcomes.

Based on our current knowledge, there are ongoing efforts of SMA study groups to update the current SMA classification to incorporate disease progression markers, marking a paradigm shift from previous disease biomarkers [31]. The concurrent results of this study further substantiate these efforts as an essential step for the field.

### 4.2 RF Models Can Provide Interpretable Predictions When Trained on Rare Disease Real‐World Data (RWD)

When considered individually, expert domain knowledge allows physicians to extract meaning from a set of otherwise non‐informative variables. Feature engineering for data representation to an ML algorithm requires physicians to rethink their approaches to RWD and convert their knowledge for the representation to an ML algorithm. In this study, we used different feature engineering methods, which included arithmetic operations and feature aggregation, to transform and select features for model training. In contrast to our approach, some algorithms have automated or intrinsic feature importance selection, e.g., neural networks and deep‐learning models that can extract meaningful features for their training. These approaches, however, require more complex models, which are often difficult to interpret, prone to hidden biases and require large amounts of data (big data). The latter condition poses an immutable challenge for a rare disease like SMA.

We used an RF model to assess the predictive power of selected features for scoliosis prediction. Previous studies have established that RFs are interpretable and robust when training on small data sets [32]. Our data set contained 750 observations derived from 84 patients (after removing scoliosis ‘NA’ labels, 65 patients with 502 observations remained). By considering individual visits and observations of clinical features at the time of visit, we achieved an accuracy of 0.82 and an average ROC of 0.87. The natural history of SMA‐associated scoliosis correlates with SMA severity. The likelihood of scoliosis development is approximately 90% in SMA Type I, 70% in SMA Type II and 40% in SMA Type III [33]. We compared the performance of our RF model to a naïve classifier that votes ‘1’ in Type I, ‘1’ in Type II and ‘0’ in Type III according to the above probabilities. The naïve classifier would achieve a 75.98% accuracy compared to our RF's 82%. Note, however, that the actual numbers are not the focus here but rather the general ability to derive meaningful information from observational data beyond already established disease markers.

### 4.3 ML Feature Selection Tools Yield Features Aligned With Expert Domain Knowledge

Another novel aspect of this study is using an ML feature selection tool, the PPS. The correlation coefficient *r* is a statistical method commonly used to find possible linear relationships between two variables. However, the correlation coefficient has its limitations [34]. One of the disadvantages of the correlation coefficient is that it is a weak predictive marker. In contrast, as an asymmetric data‐type agnostic score, the PPS can detect linear and nonlinear relationships between two variables. This is essential regarding the data types presented in the SMAScoliosis data set. The observation of PPS also allows for the inference of causality. For example, we observed that ‘height’, ‘weight’, ‘BMI’ and ‘head circumference’ had high PPS for the target label ‘scoliosis’. These variables are a function of the patient's age and developmental stage. This corresponds with our understanding of scoliosis progression in SMA, where the natural history of SMA disease includes an increased lifetime risk of scoliosis. The risk of scoliosis is a function of the patient's survival and stage of the disease.

### 4.4 Our Approach to Engineer the ‘Contracture Score’ Differs From the Historical Contracture Index (CI)

Contractures of the limbs are significant orthopaedic features that reflect the severity of neuromuscular disease (NMD) and increase the likelihood of scoliosis development in SMA patients. Factors contributing to the development of contractures in NMDs include decreased mobility, associated static positions over long periods, agonist–antagonist imbalance and fibrotic changes in the muscles [35]. Previous studies have demonstrated that contractures in SMA occur early in the knees and hips, making lower limb contractures most prevalent among SMA patients [20, 21]. Contractures of the upper limbs are often left untreated unless severe disability is present. Notably, our ‘contracture score’ is different from the relative CI that was developed by Johnson et al. in 1992 [36] to assess the risk of a contracture in neuromuscular scoliosis (NMS). The latter depends on the patient cohort and their cross‐sectional assessment. It requires experienced examiners and a goniometer to assess the passive range of motion (ROM) by neutral‐null‐method [36]. Our ‘contracture score’ contains the location of the contracture ranked by significance to SMA progression and contracture severity.

## 5 Limitations

### 5.1 SMAScoliosis Data Set Representation and Potential Confounders

SMA Type I patients are overrepresented in the SMAScoliosis data set compared to historical SMA data sets. This is partly due to new treatment options offered at our centre and positive effects on survival [7]. This patient group also represents the most understudied SMA type with the most significant potential for changes in phenotype in response to novel therapies [13]. Thus, the representation of this patient demographic is an advantage of the SMAScoliosis data set that we leveraged toward developing a prediction model. However, this currently leads to a skewed age distribution with an overrepresentation of younger age groups, which may be associated with ‘unknown’ scoliosis labels.

While the functional motor scores and disease progression markers ‘HFMSE score’, ‘6MWT’, ‘contractures score’ and ‘ventilation score’ ranked among the strongest predictors for scoliosis, the functional motor scores validated for younger patient groups, such as the CHOP‐Intend, HINE‐2 and ULF did not show high PPS. One reason could be that the scoliosis in this younger age group often remains undetected and thus is labelled ‘unknown’ in our data set. Under these circumstances, the related feature ‘age’ creates a ‘blind spot’ for the associated features to predict scoliosis. Data collection, including valid measurements of spine radiographs in this younger age group, would likely improve the predictive power in the future; however, it must be justified given the radiation exposure.

### 5.2 The Target Label ‘Scoliosis’

We labelled the onset of scoliosis using different scoliosis detection methods (Figure 2). The current gold standard for the diagnosis of scoliosis is a spinal radiograph. These were available for 41 patients. When these were unavailable, we relied on externally documented spinal radiographs in the EHR. Next, we considered chest radiographs for scoliosis detection. Studies that examined the value of posteroanterior chest radiographs in detecting scoliosis have reported up to 93.94% sensitivity for thoracic curves and a 40% sensitivity for lumbar curves [37, 38, 39]. This technique may overestimate Thoracic curves and lumbar curves may be missed. Thus, it is possible that scoliosis labels ‘0’ may have included false negatives with minor lumbar curves. We labelled ‘scoliosis’ with the clinical exam in the EHR to minimise the uncertainty. According to the current literature, detecting scoliosis based on clinical exams has variable sensitivity. It has been chiefly tested for idiopathic scoliosis, with one study reporting the reliability of physical examinations in SMA specifically [40]. We regarded higher‐grade clinical scoliosis as reliable enough for scoliosis labelling. However, minor scoliosis may have been missed due to the method's low sensitivity for lower‐grade scoliosis. In 19 patients, the scoliosis labels were set to ‘unknown’ (NA). To further explore the potential value of the ‘unknown’ data (‘missingness’), we re‐evaluated patient EHRs. We found that seven of the 19 patients were either deceased or had relocated, and four patients were below 1 year of age, thus very young to receive spine radiography. Given this additional information, future studies may also consider a multi‐class prediction approach that includes ‘unknown’ as a classifier.

### 5.3 Anticipated Changes Due to New Policies

Less than 2% of newborns are screened for SMA worldwide, with the expected increase as new data on the efficacy of SMA therapies and funding emerge [9]. SMA was implemented in Germany's country‐wide newborn screening programme in October 2021. Some of these effects can be observed in the SMAScoliosis data set, e.g., early diagnosis of presymptomatic patients who received onasemnogene abeparvovec treatment. Historically, presymptomatic SMA screening was only subjected to children with affected family members. Due to the ongoing policy and treatment strategies changes, disease progression will undergo a modification [31]. This, in turn, underscores the need for more adaptive and dynamic approaches. That is a data‐driven approach that can learn from patterns in a data set.

## 6 Conclusion

Data‐driven ML approaches in rare paediatric diseases have the potential to democratise expertise by learning from patterns in a changing disease landscape to support physicians' treatments and improve their standards of care. The introduction of disease‐modifying therapies for SMA, followed by the inclusion of SMA in newborn screenings, has presented physicians with never‐before‐seen patients and new unknowns regarding the best treatment standards. Through predictive modelling of this data, we defined disease progression markers, which are easily assessed during patient visits and relevant to both physician and ML models. Based on these markers, we could train an ML model to anticipate the onset of scoliosis. This highlights the value of progressive markers in the ongoing efforts to update the SMA classification. These findings have the potential to shift from current static markers toward advanced anticipation of scoliosis development in SMA. This new approach could allow us to provide earlier and better scoliosis treatment in a changing patient landscape. We advocate for the consistent documentation of relevant progression markers, which will serve as a basis for data‐driven models that physicians can use to update their best standard‐of‐care practices. We can direct our ongoing digitalisation processes in the clinics by building on this framework.

## Ethics Statement

This study was approved by the Charité Universitätsmedizin Berlin Ethics Committee (EA2/061/18) in 2018 and has been performed in accordance with the ethical standards laid down in the 1964 Declaration of Helsinki and its later amendments. All patients have given their informed consent before this study's analyses.

## Conflicts of Interest

Claudia Weiß is on the honorary advisory board of Novartis, Roche and Biogen and has given an honorary presentation at conferences for Novartis. The other authors declare no conflicts of interest.

## Supporting information


**Figure S1.** Grouped non‐engineered features in the SMAScoliosis data set.

**Figure S2.** PPS ranking for individual items of the functional motor scores.

**Figure S3.** PPS ranking for individual items of the functional motor scores.

**Table S1.** Random Forest Classifier Performance Metrics across ten groupedKFold cross‐validation runs
