# Supplementary material for: Feature Engineering for the Prediction of Scoliosis in 5q‐Spinal Muscular Atrophy
Source: J Cachexia Sarcopenia Muscle. 2024 Dec 5;16(1):e13599. doi: 10.1002/jcsm.13599 (PMC11670177; doi:10.1002/jcsm.13599)
Supplement: Supplementary file 1 — Figure S1. Grouped non‐engineered features in the SMAScoliosis data set. [file JCSM-16-e13599-s003.pdf]

S-Fig 1

'Demographic'

sex

'female'
'male'
'undifferentiated'
'unknown'

'SMA markers'

sma_type

'type 0'
'type 1'
'type 2'
'type 3'
'type 4'
'unknown'

smn2_copies

'0'
'1'
'2'
'3'
'4'
'5'
'6'
'7'
'8'
'>8'
'unknown'

'SMA markers II'

smn1_mut_type

'none'
'homozygous_delex7'
'homozygous_delex8'
'compound_heterozyg_mut'
'smn1_point_mut'
'complete_del_smn1'
'other'
'unknown'

'Assessment'

age_assessment

calculated date field

gastric_tube

'none'
'yes, suppl. fluids'
'yes, excl. by tube'

clinical_exam

height
height percentile
weight
bmi
head_circumference
head_circumference_perc

clinical_exam

cx_skin
cx_heent
cx_pulmo
cx_heart
cx_abdomen
cx_neurology

'Motor Skills'

upper_limb_function

'ulf_1'
'ulf_2'
'ulf_3'

hine_motor_score

hine_1
hine_2
hine_3
hine_4
hine_5
hine_6
hine_7
hine_8

'Motor Scores'

chop_motor_score

chop_1
chop_2
chop_3
chop_4
chop_5
chop_6
chop_7
chop_8
chop_9
chop_10
chop_11
chop_12
chop_13
chop_14
chop_15
chop_16

6mwt_score

meters

'Motor Scores II'

rulm_score

rulm_1
rulm_2
rulm_3
rulm_4
rulm_5
rulm_6
rulm_7
rulm_8
rulm_9
rulm_10
rulm_11
rulm_12
rulm_13
rulm_14
rulm_15
rulm_16
rulm_17
rulm_18
rulm_19

'Motor Scores III'

hfmse_score

hfmse_1
hfmse_2
hfmse_3
hfmse_4
hfmse_5
hfmse_6
hfmse_7
hfmse_8
hfmse_9
hfmse_10
hfmse_11
hfmse_12
hfmse_13
hfmse_14
hfmse_15
hfmse_16
hfmse_17
hfmse_18
hfmse_19
hfmse_20

hfmse_score20

hfmse_21
hfmse_22
hfmse_23
hfmse_24
hfmse_25
hfmse_26
hfmse_27
hfmse_28
hfmse_29
hfmse_30
hfmse_31
hfmse_32
hfmse_33

hfmse_score33
