# Supplementary figures and images for: Feature Engineering for the Prediction of Scoliosis in 5q‐Spinal Muscular Atrophy
Source: J Cachexia Sarcopenia Muscle. 2024 Dec 5;16(1):e13599. doi: 10.1002/jcsm.13599 (PMC11670177; doi:10.1002/jcsm.13599)

**S-Fig. 1**

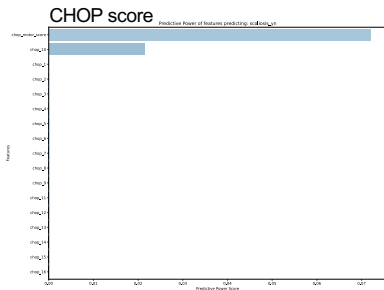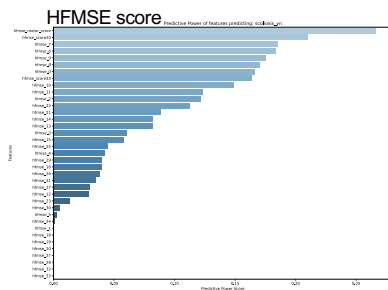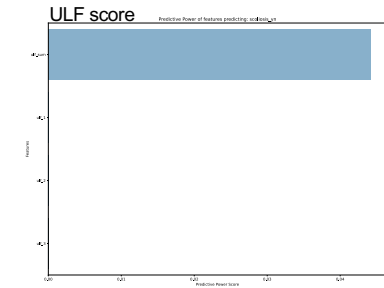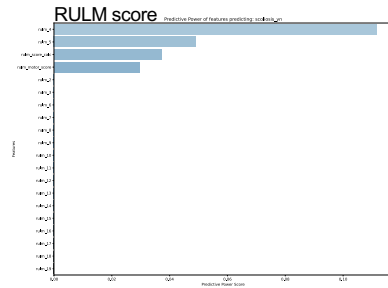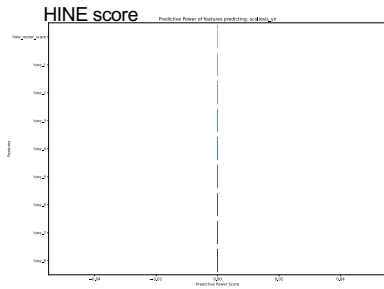

Supplement: Supplementary file 2 — Figure S2. PPS ranking for individual items of the functional motor scores. [file JCSM-16-e13599-s002.pdf]

S-Fig 3

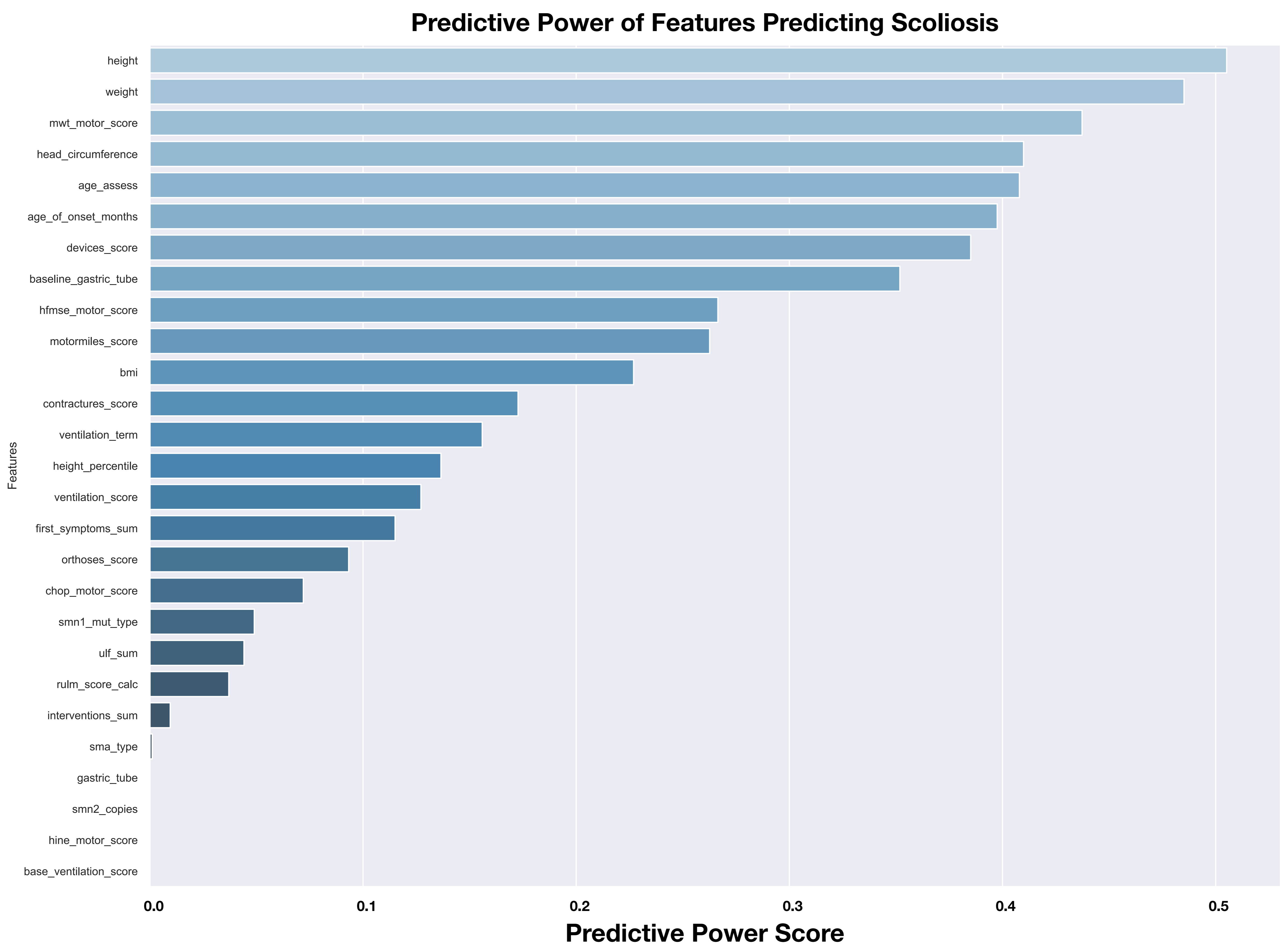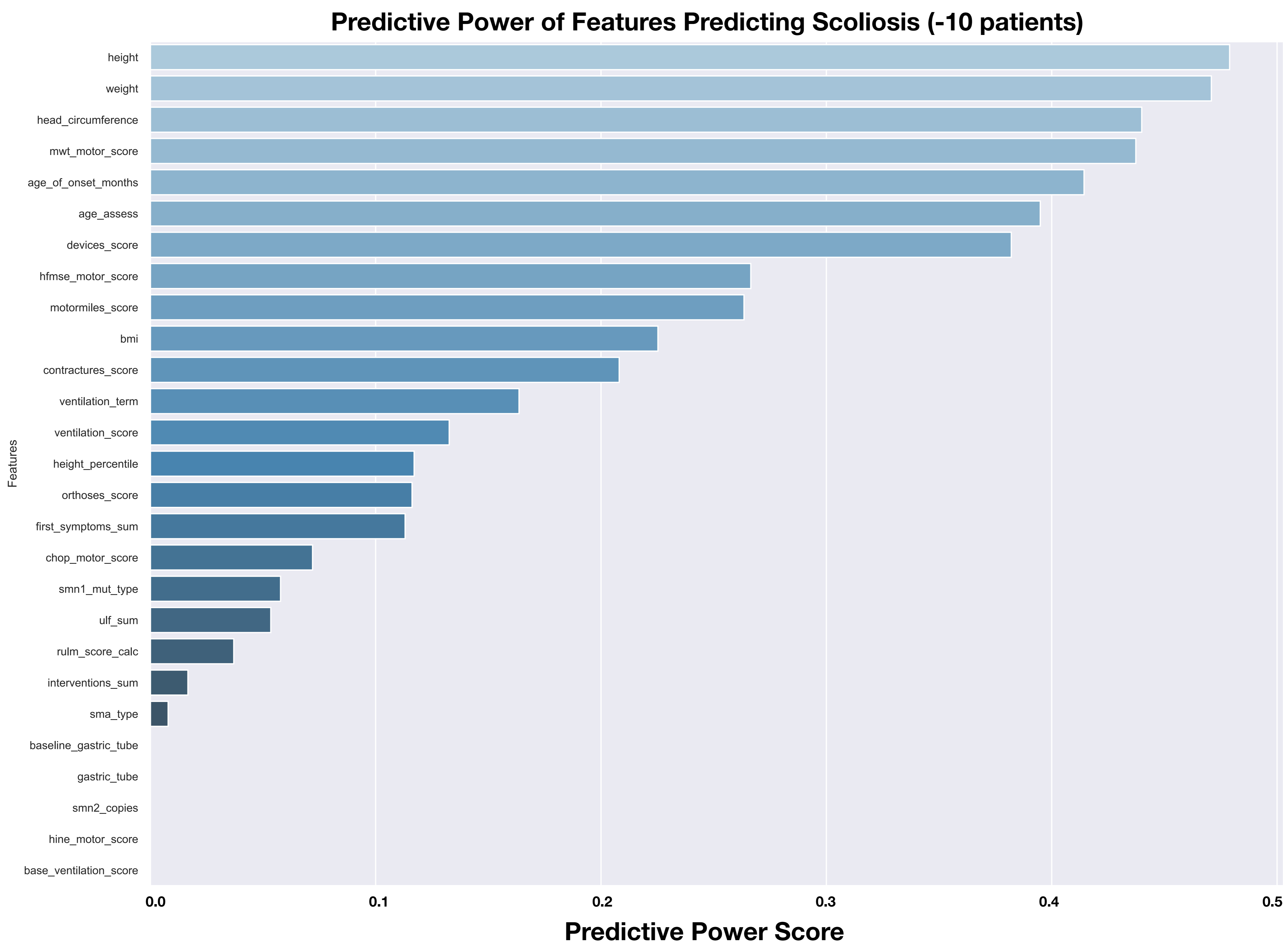

Supplement: Supplementary file 3 — Figure S3. PPS ranking for individual items of the functional motor scores. [file JCSM-16-e13599-s004.pdf]
