# Supplementary material for: Feature Engineering for the Prediction of Scoliosis in 5q‐Spinal Muscular Atrophy
Source: J Cachexia Sarcopenia Muscle. 2024 Dec 5;16(1):e13599. doi: 10.1002/jcsm.13599 (PMC11670177; doi:10.1002/jcsm.13599)
Supplement: Supplementary file 4 — Table S1. Random Forest Classifier Performance Metrics across ten groupedKFold cross‐validation runs [file JCSM-16-e13599-s001.pdf]

Supplemental Table 1

Fold	Precision	Recall	F1-Score	Accuracy	ROC AUC
**1**	0.89	0.89	0.89	0.893	0.94
**2**	0.88	0.88	0.88	0.88	0.89
**3**	0.81	0.73	0.73	0.746	0.86
**4**	0.82	0.74	0.71	0.739	0.73
**5**	0.88	0.84	0.83	0.84	0.84
**6**	0.82	0.81	0.81	0.808	0.88
**7**	0.84	0.81	0.81	0.81	0.84
**8**	0.8	0.78	0.77	0.778	0.92
**9**	0.86	0.86	0.86	0.86	0.85
**10**	0.87	0.86	0.86	0.86	0.94
**Average**	0.847	0.82	0.815	0.8214	0.869
